# The Use of Nanoscale Visible Light-Responsive Photocatalyst TiO_2_-Pt for the Elimination of Soil-Borne Pathogens

**DOI:** 10.1371/journal.pone.0031212

**Published:** 2012-02-22

**Authors:** Ya-Lei Chen, Yao-Shen Chen, Hao Chan, Yao-Hsuan Tseng, Shu-Ru Yang, Hsin-Ying Tsai, Hong-Yi Liu, Der-Shan Sun, Hsin-Hou Chang

**Affiliations:** 1 Department of Biotechnology, National Kaohsiung Normal University, Kaohsiung, Taiwan; 2 Division of Infectious Diseases, Kaohsiung Veterans General Hospital, Kaohsiung, Taiwan; 3 Graduate Institute of Medical Science, Tzu-Chi University, Hualien, Taiwan; 4 Department of Chemical Engineering, National Taiwan University of Science and Technology, Taipei, Taiwan; 5 Department of Molecular Biology and Human Genetics, Tzu-Chi University, Hualien, Taiwan; 6 Department of Internal Medicine, National Yung-Ming University, Taipei, Taiwan; Indian Institute of Science, India

## Abstract

Exposure to the soil-borne pathogens *Burkholderia pseudomallei* and *Burkholderia cenocepacia* can lead to severe infections and even mortality. These pathogens exhibit a high resistance to antibiotic treatments. In addition, no licensed vaccine is currently available. A nanoscale platinum-containing titania photocatalyst (TiO_2_-Pt) has been shown to have a superior visible light-responsive photocatalytic ability to degrade chemical contaminants like nitrogen oxides. The antibacterial activity of the catalyst and its potential use in soil pathogen control were evaluated. Using the plating method, we found that TiO_2_-Pt exerts superior antibacterial performance against *Escherichia coli* compared to other commercially available and laboratory prepared ultraviolet/visible light-responsive titania photocatalysts. TiO_2_-Pt-mediated photocatalysis also affectively eliminates the soil-borne bacteria *B. pseudomallei* and *B. cenocepacia*. An air pouch infection mouse model further revealed that TiO_2_-Pt-mediated photocatalysis could reduce the pathogenicity of both strains of bacteria. Unexpectedly, water containing up to 10% w/v dissolved soil particles did not reduce the antibacterial potency of TiO_2_-Pt, suggesting that the TiO_2_-Pt photocatalyst is suitable for use in soil-contaminated environments. The TiO_2_-Pt photocatalyst exerted superior antibacterial activity against a broad spectrum of human pathogens, including *B. pseudomallei* and *B. cenocepacia*. Soil particles (<10% w/v) did not significantly reduce the antibacterial activity of TiO_2_-Pt in water. These findings suggest that the TiO_2_-Pt photocatalyst may have potential applications in the development of bactericides for soil-borne pathogens.

## Introduction

Upon ultraviolet (UV) light illumination, a traditional photocatalyst generates pairs of electrons and holes (electron vacancies in valence bands) to yield reactive oxygen species (ROS) [1], which can oxidize organic substances and kill pathogenic bacteria [2]. Titania photocatalysts have great potential for use in water and sewage treatment because they are stable in water, non-toxic by ingestion and inexpensive [2]. Because the energy source can be solar light, TiO_2_ photocatalysts are also useful in remote areas where sufficient electricity is not available. To obtain higher quantum efficiencies and reduce the potential exposure of humans and animals to bio-hazardous UV light, ion-doped TiO_2_ materials with improved visible light responsiveness have recently been developed [3], [4]. These photocatalytic materials have different degrees of bactericidal properties [5]–[10]. Despite their advantages, the elimination of soil-borne pathogens using UV and visible light-responsive photocatalysts has not been clearly addressed.

This study focused on *Burkholderia pseudomallei* and *Burkholderia cenocepacia*, two soil-borne pathogens that can cause the fatal infectious diseases melioidosis and cepacia syndrome, respectively [11], [12]. Humans are usually infected with these pathogens through inhalation or cutaneous contact with contaminated soil or water [11], [13]–[16]. These two pathogens are both motile, rod-shaped, Gram-negative bacteria, but they also exhibit certain distinct features. *B. cenocepacia* is widely distributed in the natural environment but is also found in hospitals. This may explain the spread of *B. cenocepacia* that sometimes occurs in intensive care units or oncology wards [14], [17], [18]. However, *B. cenocepacia* species primarily participate in non-hazardous interactions with plants. Some of these bacteria are even beneficial to humans because they can produce biosurfactants that increase the solubility of pesticides present in polluted environments or provide effective defenses for crop plants against the pathogenic fungus *Fusarium verticillioides* [19], [20]. These benefits, however, may increase incidental human contact. By contrast, *B. pseudomallei* mainly dwell in the soil at a depth of up to 60 cm below the soil surface. Thus, outbreaks of *B. pseudomallei* usually occur after seasonal events, such as monsoons or typhoons [11], [13], [21], [22], although *B. pseudomallei* still affects millions of people in many countries annually [23], [24]. Because *B. pseudomallei* is a potent infectious agent, aerosol exposure during an intentional attack remains a concern [25]. Many clinical isolates of *B. pseudomallei* can tolerate a wild spectrum of antibiotics/bactericides, including penicillin, first- and second-generation cephalosporins and many of the aminoglycosides [16]. Similarly, *B. cenocepacia* are intrinsically resistant to most clinically relevant antibiotics such as quinolones, aminoglycosides and β-lactam agents, including monobactams and carbapenems [15], [26]. Unfortunately, there are still no licensed vaccines available for these pathogens. Because both bacteria steadily adhere to soil particles, resulting in the transmission of disease when the bacteria are aerosolized [12], [13] or contaminate soil and water [13], [14], effective control strategies to overcome the spread of these bacteria need to be developed.

A visible light-responsive titania photocatalyst is a conceptually feasible candidate for an antibacterial approach because it combines the advantages of a titania photocatalyst in water and sewage treatment [2] with the user-friendliness of visible light [5]–[7]. To select a high-performing visible light-responsive photocatalyst, commercially available and laboratory-prepared photocatalysts were evaluated for photocatalysis-mediated antibacterial activity against *Escherichia coli*. TiO_2_-Pt nanoparticles enable superior photocatalytic degradation of pollutant nitrogen oxides [27], and this study demonstrated that, among our selected photocatalysts, TiO_2_-Pt nanoparticles also exhibit superior antibacterial activity. Therefore, the antibacterial activity of TiO_2_-Pt nanoparticles against *B. pseudomallei* and *B. cenocepacia* in suspended cultures and/or biofilms was further evaluated under visible light illumination. The attenuation of soil-borne pathogens by TiO_2_-Pt-mediated photocatalysis was also investigated in a mouse model. We unexpectedly found that the TiO_2_-Pt photocatalyst continued to exhibit superior antibacterial activity in soil-contaminated water. The potential mechanisms are discussed.

## Results

### Antibacterial activity of various titania photocatalysts

**Figure 1:**
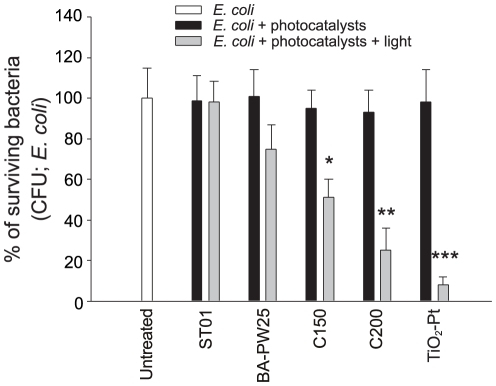
Antibacterial activity of nanoscale TiO_2_-Pt. The antibacterial activity of TiO_2_-Pt-mediated photocatalysis against *E. coli* is compared with other UV and visible light-responsive photocatalysts. The bacterial number (CFU) in the untreated groups was normalized to 100%. * *P*<0.05, ** *P*<0.01 and *** *P*<0.001, compared to the respective groups without light. n = 6 (3 experiments with 2 replicates). The data are presented as mean ± SD.

Under visible light illumination, the nanoscale TiO_2_-Pt samples exerted superior killing of *E. coli* [5], [10] compared to the commercially available BA-PW25 [28], [29] and carbon-containing TiO_2_ (C150, C200) [7], [9], [30] (Fig. 1, * *P*<0.5, ** *P*<0.01 and *** *P*<0.001, compared to the respective without light groups). The ultraviolet (UV) light-responsive photocatalyst (ST01) was used as a negative control because it does not respond to visible light illumination (Fig. 1, ST01 groups) [7], [27]. To further investigate the bactericidal spectrum of TiO_2_-Pt nanoparticles, various human pathogens, including different strains of the soil-borne bacteria *B. pseudomallei* and *B. cenocepacia*, were analyzed (Table 1). TiO_2_-Pt-mediated photocatalysis eliminated a wide spectrum of human pathogens. Among these pathogens, *B. pseudomallei* strains were the most susceptible to TiO_2_-Pt-mediated photocatalysis (Table 1, *B. pseudomallei* vgh07, vgh19, vgh21; 15–18% survival rates).

**Table 1:** The antibacterial spectrum of the nanoscale TiO_2_-Pt photocatalyst.

Species and strains	Survival (%)
**Nonpathogenic bacteria**	
*Escherichia coli*	
OP50	9±4
**Pathogenic bacteria**	
**Soil-borne**	
*Burkholderia cenocepacia*	
P2	26±3
34B	47±2
BC14	60±1
*Burkholderia pseudomallei*	
vgh07	17±8
Vgh19	15±4
Vgh21	18±3
**Non-soil-borne**	
*Staphylococcus aureus*	
ATCC6538P	30±5
Multidrug resistant, strain 27	45±1
Multidrug resistant, strain 69	51±7
Coagulase negative, strain 41	24±5
*Acinetobacter baumannii*	
nknu11	30±4
Multidrug resistant, strain 49	40±4
*Legionella pneumophila*	
ATCC33152	37±3
*Pseudomonas aeruginosa*	
FY32	48±7
*Klebsiella pneumoniae*	
nknu24	34±2
*Salmonella typhimurium*	
FYI48	41±7

The survival rate of various bacteria after challenged with TiO_2_-Pt-mediated photocatalysis. The visible light-driven antibacterial activity of TiO_2_-Pt-mediated photocatalysis against various bacteria is shown; the soil-borne pathogens *B. pseudomallei* and *B. cenocepacia* were compared with nonpathogenic *E. coli* OP50 and the pathogenic bacteria *S. aureus*, *A. baumannii*, *L. pneumophila*, *P. aeruginosa*, *K. pneumoniae* and *S. typhimurium*. The untreated groups (without TiO_2_-Pt and illumination) of each experiment were normalized to 100%. n = 6, three experiments with 2 replicates).

### Attenuation of *B. pseudomallei* and *B. cenocepacia* by TiO_2_-Pt-mediated photocatalysis

**Figure 2:**
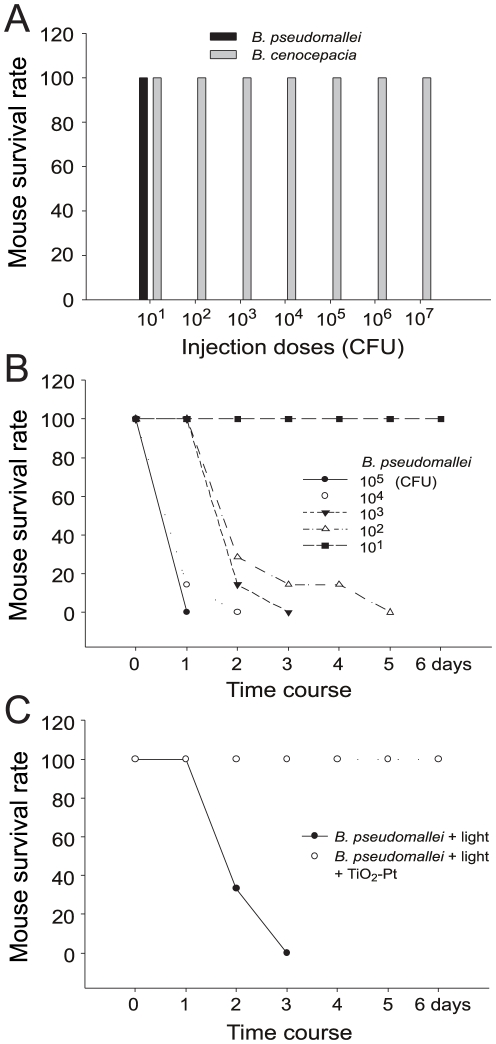
Mortality of mice receiving inoculations of *B. pseudomallei* with or without photocatalysis. The survival rate of mice receiving inoculations of various doses (10^1^–10^6^) of *B. pseudomallei* vgh07 and *B. cenocepacia* P2 cells is shown. n = 7, three experiments with 2 or 3 replicates (A). The survival rate and time course of mice treated with 10^1^–10^6^ CFU of *B. pseudomallei* vgh07. n = 7, three experiments with 2 or 3 replicates (B). The survival rate and time course of mice treated with 10^3^ CFU of *B. pseudomallei* vgh07 with or without TiO_2_-Pt -mediated photocatalysis. n = 6, three experiments with 2 replicates (C).

In addition to the killing effect, our previous works indicated that photocatalysis introduced cellular damages to those survivors also plays an important role in the attenuation of pathogenic bacteria [6]. Accordingly, we hypothesized that the viable populations of photocatalyzed *B. pseudomallei* vgh07 or *B. cenocepacia* P2 (survival rates of 17% and 26% in Table 1, respectively) would have reduced pathogenic potency. To investigate this possibility, the lethal doses of *B. pseudomallei* vgh07 and *B. cenocepacia* P2 in mice were first determined. A single inoculation of 1×10^2^ CFU of *B. pseudomallei* vgh07 resulted in 100% mortality (Fig. 2A, 2B, *B. pseudomallei* 10^2^ CFU groups). By contrast, up to 1×10^7^ CFU of *B. cenocepacia* P2 was unable to induce mortality in BALB/cJ mice (Fig. 2A). Because small changes in bacterial doses cause a dramatic difference in mortality, *B. pseudomallei* infection in mice should be a more sensitive model than *B. cenocepacia* for investigating photocatalysis-induced attenuation. However, because the range of sublethal doses is narrow (<10^2^ CFU), the combined effect of photocatalysis and the host immune defenses easily eliminates the injected *B. pseudomallei*, and thus the attenuation of inflammation is not easily observed. Alternatively, clinical features of cepacia syndrome, such as leukocytosis, inflammation and liver necrosis, were clearly reproduced in BALB/cJ mice to a certain extent using a sublethal dose of *B. cenocepacia* (10^5^ CFU/mouse). Thus, the bacteria *B. pseudomallei* and *B. cenocepacia* were used to investigate the potential attenuating effects on mortality and the inflammatory response, respectively.

In the mouse model, TiO_2_-Pt-mediated photocatalysis significantly attenuated the lethal dose of *B. pseudomallei* vgh07 treatments (1×10^3^ CFU; Fig. 2A, 2B, 100% mortality) and resulted in a 100% survival rate for the infected mice (Fig. 2C, TiO_2_-Pt groups). Notably, the TiO_2_-Pt-photocatalysis groups in which there was no mortality (Fig. 2C, TiO_2_-Pt groups) were apparently equivalent to treatment with 1×10^1^ CFU of *B. pseudomallei* without photocatalysis (Fig. 2A, 2B, *B. pseudomallei*, 10^1^ groups). According to the survival rate estimated in the bacterial killing experiments (Table 1, 17%, *B. pseudomallei* vgh07 groups), approximately 1.7×10^2^ CFU of bacterial cells should theoretically remain viable (1×10^3^ CFU×17% = 1.7×10^2^ CFU), which is still a lethal dose for BALB/cJ mice (Fig. 2A, 2B, 1×10^2^ CFU *B. pseudomallei* groups, 100% mortality). This suggests that the TiO_2_-Pt photocatalyzed bacteria are greatly attenuated, similar to our previous study in which anthrax spores were used as a model system [6]. One possible explanation is that the reduction in the pathogenicity of photocatalyzed *B. pseudomallei* is due to the combination of the reduction in viable cells and the damage to the surviving cells. It has been suggested that, although photocatalysis-induced damages may be repaired in culture, the bacteria may not be recoverable due to the stress of host phagocytic clearance, thus causing differences in *in vitro* and *in vivo* analyses [6], [9]. Accordingly, it is estimated that, in addition to bacterial killing, at least a one-log reduction in the pathogenic potency of *B. pseudomallei* can be attributed to bacterial damage (estimated 1.7×10^2^ CFU viable cells vs. 100% survival rates in Fig. 2C, TiO_2_-Pt groups, and Fig. 2A, 10^1^ groups).

**Figure 3:**
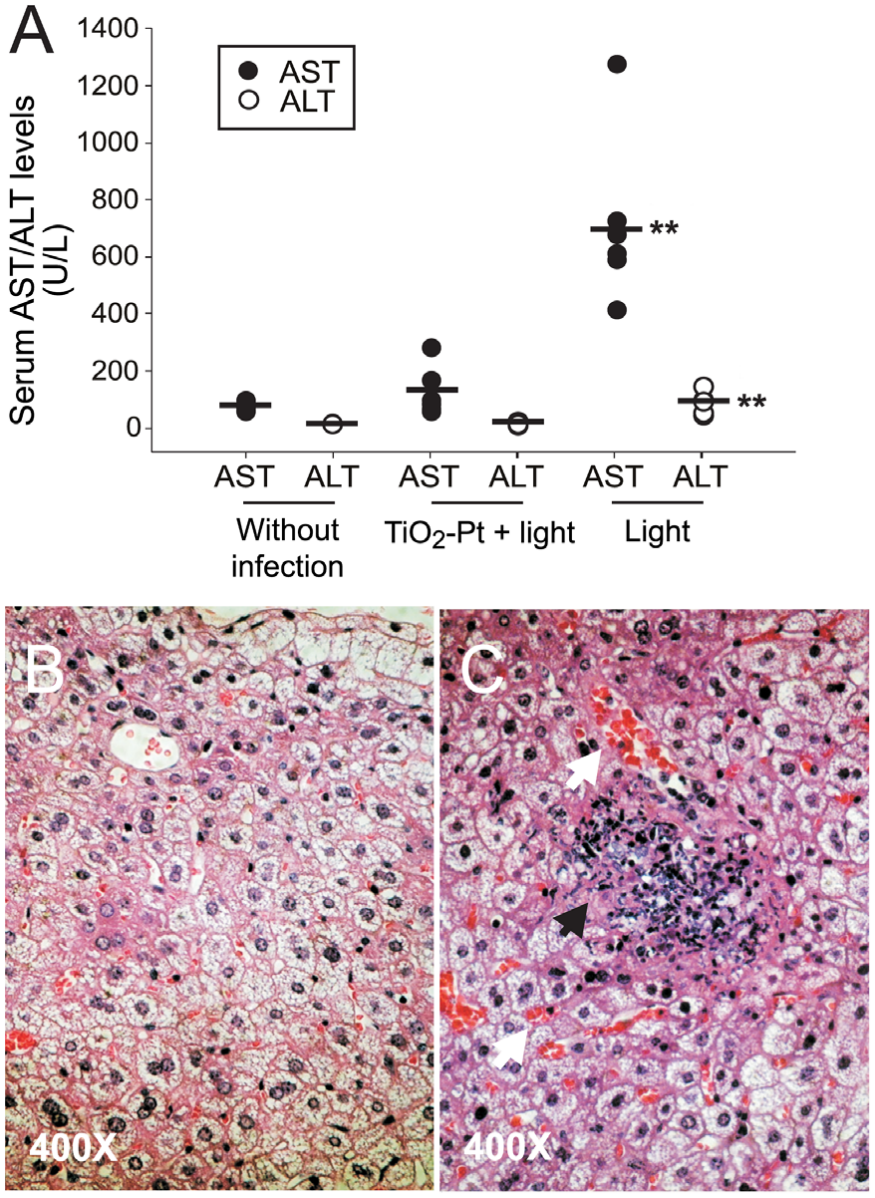
Liver damage in mice infected with *B. pseudomallei*. Twenty-four hours after inoculation with *B. pseudomallei* with or without photocatalysis, the serum AST/ALT levels of mice were examined. The means of the respective groups are indicated as horizontal bars; ** *P*<0.01 and **†††** *P*<0.001, compared with the TiO_2_-Pt+light photocatalyzed groups and without infection groups, respectively (A). The hematoxylin and eosin staining of liver sections from mice treated with *B. pseudomallei* with (B) or without (C) photocatalysis. White arrows: hemorrhage lesions; black arrow: cellular fragment and debris. n = 6, three experiments with 2 replicates.

Melioidosis frequently manifests in the formation of abscesses in soft tissues and internal organs like the liver [11]. Hepatic cellular debris in the liver has been reproduced in mice that have received an intravascular infection of *B. pseudomallei* [31]. Therefore, in this study, the liver function of mice served as an indicator of disease severity and was evaluated by analyzing the plasma levels of the hepatocyte enzymes aspartate aminotransferase/alanine aminotransferase (AST/ALT), which are markers of liver function (Fig. 3A). In agreement with the mortality data (Fig. 2C), the induction of plasma AST/ALT was significantly reduced when BALB/cJ mice were infected with photocatalyzed *B. pseudomallei* vgh07 (Fig. 3A, TiO_2_-Pt+light vs. light groups, ** *P*<0.01). Similarly, lesions with cellular debris in the liver were not found in these photocatalyzed groups (Fig. 3B vs. 3C; white arrows: hemorrhage lesions; black arrow: cellular fragmentations and debris [31]).

### Attenuation of *B. cenocepacia*-mediated inflammation by photocatalysis

**Figure 4:**
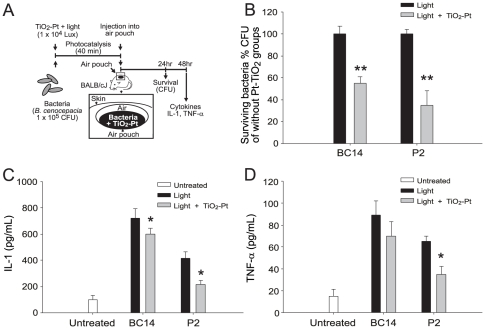
Bacterial survival and inflammatory cytokine production in mice. The experimental outline of the air pouch infection mouse model is shown (A). The survival rate of *B. cenocepacia* cells (strains BC14 and P2), which were treated with or without photocatalysis, was recorded 24 hours after subcutaneous injection into mice. ** *P*<0.01, compared to the respective without-photocatalyst groups (B). The levels of the inflammatory cytokines IL-1 (C) and TNF-α (D) in the air pouches underneath the mouse skin were also determined. * *P*<0.05, compared to the respective without-photocatalyst groups. n = 6, three experiments with 2 replicates. The data are presented as mean ± SD.

To investigate whether photocatalysis could attenuate *B. cenocepacia* and thus result in reduced inflammation *in vivo*, an air pouch infection mouse was established based on a previously described method [32]. *B. cenocepacia* cells (strains P2 and BC14; 1×10^5^ CFU) were treated with or without TiO_2_-Pt-mediated photocatalysis (visible light, 1×10^4^ lux) and then injected into air pouches underneath the skin of mice (Fig. 4A, the experiment outline). Twenty-four hours post treatment, viable bacteria were recovered from the air pouch (Fig. 4A, experiment outline). In agreement with the *in vitro* analysis (Table 1, P2 and BC14 groups), photocatalysis was associated with significantly fewer viable bacteria (Fig. 4B, ** *P*<0.01, compared with the respective light-only/without TiO_2_-Pt groups).

Forty-eight hours after the injection of *B. cenocepacia* P2 and BC14, which allowed the infection to become established in the internal organs, the serum levels of the cytokines interleukin-1 (IL-1) and tumor necrosis factor-α (TNF-α) were characterized (Fig. 4A, experiment outline; Fig. 4C–D). In agreement with the analyses performed *in vitro* (Table 1, P2 and BC14 groups) and *in vivo* (Fig. 4B), the photocatalyzed *B. cenocepacia* caused a significantly lower induction of IL-1 and TNF-α than bacteria that were not treated with photocatalysis (Fig. 4C, 4D; light vs. light+TiO_2_-Pt groups; * *P*<0.05, ** *P*<0.01). These results suggest that photocatalysis reduced *B. cenocepacia-*mediated inflammatory responses in mice.

### Photocatalysis resistance associated with biofilm mass

**Figure 5:**
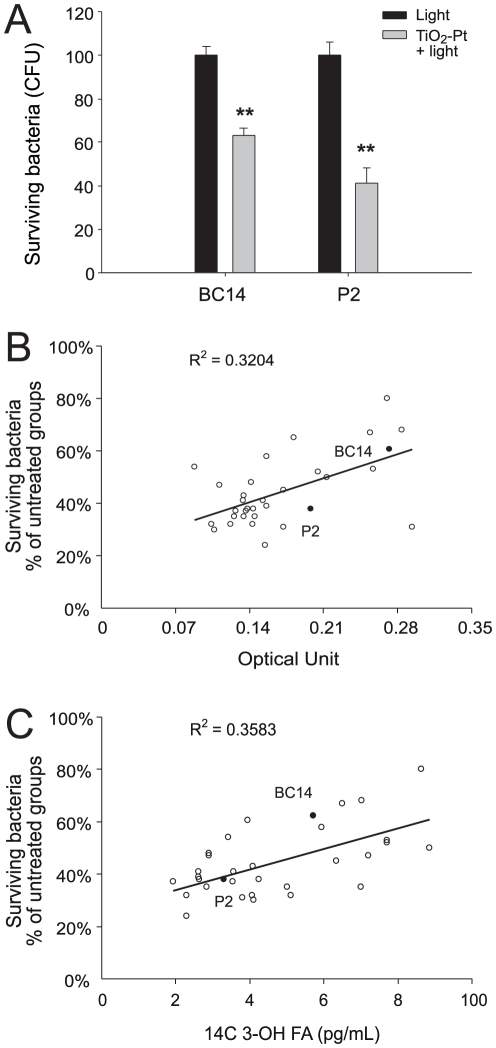
Association between biofilm formation and resistance to photocatalysis. The survival rate of *B. cenocepacia* cells (strains BC14 and P2) in biofilms after the photocatalysis treatments is shown (A). The data are presented as mean ± SD. Correlations between the bacterial survival rates of 30 different *B. cenocepacia* isolates and their ability to form biofilms are indicated; optical unit: the residual crystal violet in the biofilm, which represents relative biofilm mass (B). The correlations between the bacterial survival rates and the medium lipopolysaccharide (LPS) levels are indicated; [C14:0 3-OH FA]: an indicator of LPS, which represents relative LPS levels (C). n = 6, three experiments with 2 replicates.

*B. cenocepacia* was relatively more resistant to photocatalysis than *B. pseudomallei* (Table 1). Photocatalysis-resistant bacteria have not been clearly characterized. The broad resistance spectrum of *B. cenocepacia* isolates may be useful for investigating the mechanism underlining photocatalytic resistance. Previous reports have indicated that biofilm formation is associated with bacterial resistance to antibiotics and the ROS hydrogen peroxide [33]–[37]. Consequently, bacterial killing experiments were performed with *B. cenocepacia* on biofilms. Biofilm formation tended to increase the survival rate of photocatalyzed *B. cenocepacia* cells (Fig. 5A, P2, 40% survived>Table 1, P2, 26% survived). To further investigate whether the ability to form a biofilm is associated with photocatalysis resistance, the survival rates of the photocatalyzed bacteria (including a total of 33 *B. cenocepacia* isolates, in which the P2 and BC14 data were equivalent to Table 1; the Y-axes of Fig. 5B and Fig. 5C) were plotted against the relative masses of the biofilms (quantified in optical units, the X-axis of Fig. 5B) and the released levels of lipopolysaccharide (LPS) (an indicator that is associated with biofilm formation ability [38]; the X-axis of Fig. 5C;). These results suggest that biofilm formation is somewhat associated with resistance to photocatalysis (Fig. 5B–C), although the detailed mechanism remains to be investigated further.

### Scanning electron microscopy

**Figure 6:**
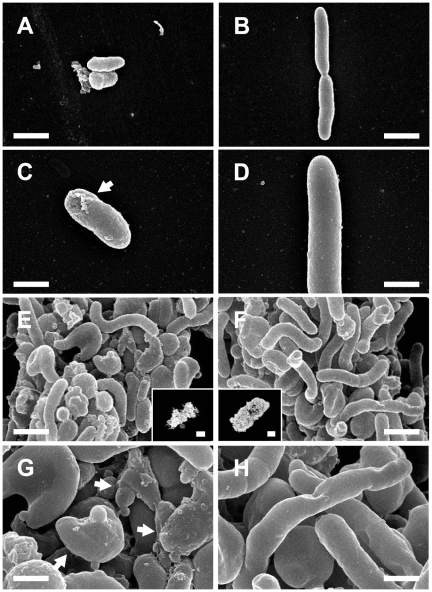
Scanning electron microscopy (SEM). SEM analysis was performed to investigate the photocatalysis-induced deformation of bacterial cells. SEM images of *B. cenocepacia* cells (strains P2: A–D and BC14: E–H) with (A, C, E, G) or without (B, D, F, H) photocatalysis were taken. Arrows in C and G indicate the cellular deformations. Inserts in E and F are the respective low magnification views of biofilm-like bacterial clusters. Scale bars: 5 µm in A, B, E, F; 2.5 µm in C, D, G, H; 10 µm in the insets of E and F.

Previous studies have suggested that photocatalysis-induced damage is crucial for the attenuation of bacterial cells [6]. Scanning electron microscopy was used to determine whether photocatalysis induced different deformations of *B. cenocepacia* strains that exhibited different degrees of resistance to photocatalysis (Fig. 6, P2 vs. BC14; P2: A–D; BC14: E–H). The bacteria were treated with (Fig. 6 A, C, E, G) or without (Fig. 6 B, D, F, H) photocatalysis. Cellular deformations were observed in the groups with photocatalysis, indicating that the bacteria were damaged (Fig. 6 A, C, E, G; arrows in C and G). Intriguingly, the P2 strain of *B. cenocepacia* was mainly present as planktonic cells (>90%; Fig. 6, A–D). By contrast, BC14 cells mainly gathered in cell clusters, which resembled miniature biofilms (>90%; Fig. 6, E–H; the small inserts in E and F are the respective low magnification views). This is the first SEM observation of biofilm-like cell clusters of *B. cenocepacia* in a suspended liquid culture. Given that biofilms can resist various bactericides, including ROS [33]–[37], and that BC14 cells have a stronger tendency to form biofilms than P2 cells (Fig. 5B–C, BC14 vs. P2; Fig. 6E–H vs. 6A–D), it is reasonable to conclude that BC14 cells have a higher resistance to photocatalysis than P2 cells (Table 1, BC14 vs. P2).

### Antibacterial performance of TiO_2_-Pt in soil-containing solutions

**Figure 7:**
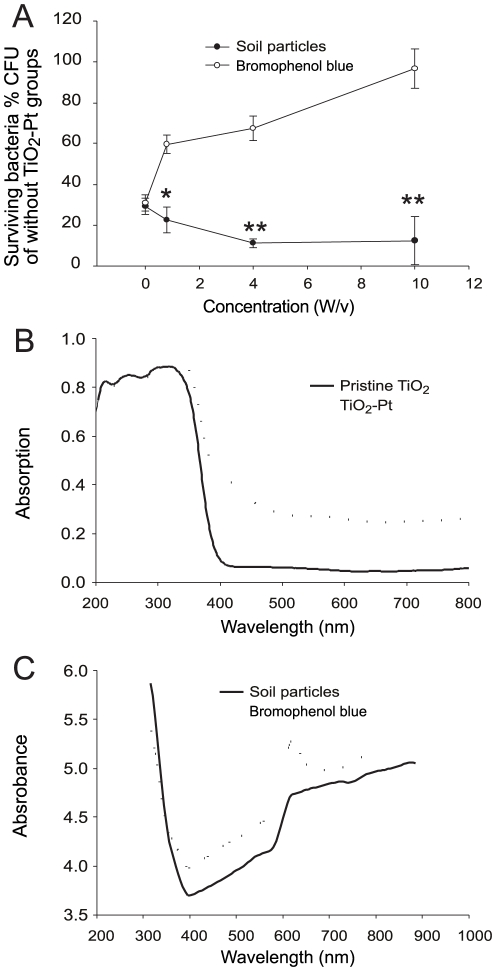
The antibacterial activity of the TiO_2_-Pt photocatalyst in soil-containing solutions. The survival rates of *B. cenocepacia* cells (strains P2) in different concentrations of soil particle and bromophenol blue solutions are shown (A). The data are presented as mean ± SD. * *P*<0.05 and ** *P*<0.01, compared with the respective bromophenol blue groups. The UV-visible light absorption spectra of TiO_2_-Pt and pristine TiO_2_ are shown (B). The absorbance of 10% w/v soil- and bromophenol blue-containing solutions at various wavelengths is indicated (C). n = 6, three experiments with 2 replicates.

One practical problem in the elimination of soil-borne pathogens is the potential light-shading effect of contaminated soil particles. Contaminants such as dye (bromophenol blue) and protein (bovine serum albumin) have been shown to greatly reduce the antibacterial activity of a photocatalyst [5]. To investigate the potential light-shading effect of soil particles, photocatalysis-mediated killing in a soil-containing solution was compared with photocatalysis-mediated killing in solutions of various concentrations of bromophenol blue (Fig. 7A). Unexpectedly, soil particle contaminants up to a concentration of 10% w/v did not significantly influence the elimination of *B. cenocepacia* P2 cells, compared to the strong blocking effect of bromophenol blue at the same dose (10% w/v; Fig. 7A, ** *P*<0.01, compared with the respective bromophenol blue groups). Similar results were also obtained using another *B. cenocepacia* strain (BC14) and *B. pseudomallei* (data not shown). One possibility is that bromophenol blue, but not the soil solution, blocks certain ranges of visible light that are vital for the activation of TiO_2_-Pt. The UV-visible light absorption spectrum of photocatalyst was therefore examined; the light absorption of TiO_2_-Pt but not pristine TiO_2_-Pt covers the entire visible light range (Fig. 7B, TiO_2_-Pt vs. pristine TiO_2_-Pt). To investigate whether the different antibacterial outcomes were due to the distinct light-absorbing properties of soil particles and bromophenol blue, the UV-visible absorption spectrum of each solution was determined (Fig. 7C, the concentration of both solutions was 10% w/v). The results indicated that there were only small differences between the light-absorbing properties of the two solutions (Fig. 7C), suggesting that the dramatically different antibacterial outcomes were not primarily due to differences in light absorption.

Another explanation is that the soil particles used in this study might affect bacterial survival. Because soil is the natural habitat of *B. cenocepacia*, soil particles would not be expected to inhibit the bacteria. Nonetheless, to test the specific soil that we used, the growth constants for *B. cenocepacia* were determined in various culture conditions, including one with soil-particle supplements (Table 2). As expected, soil particles did not suppress the growth of *B. cenocepacia* (Table 2, soil media vs. LB and BCS media). Collectively, these results suggest that TiO_2_-Pt is useful for eliminating pathogens in soil-contaminated water. The differential blocking of photocatalysis by bromophenol blue and soil-containing solutions is interesting, and its mechanism remains to be further investigated.

**Table 2:** The specific growth rate constant (k) of *B. cenocepacia* P2 under various culture conditions.

Specific growth rate constant (k) of *B. cenocepacia* P2: lnN0-lnN1 = k (t0–t1) k>0 growth; k = 0 critical point; k<0 decline.			
Culture media	Exp 1	Exp 2	Exp 3
**LB broth**	0.67	0.61	0.66
**Soil media**	0.60	0.62	0.64
**BCS media**	0.66	0.64	0.61

The specific growth rate constant (k) was measured after growing *B. cenocepacia* P2 in LB broth, soil medium (soil particles 10% w/v in LB) and *B. cepacia* selective (BCS) medium.

## Discussion

Traditional UV-responsive photocatalysts have shown great potential in the development of a new generation of disinfectants [1]. Because UV light can damage human eyes and skin [39], [40], photocatalysts with improved visible light responsiveness have been developed [3]–[7]. The antibacterial activity of a visible light-responsive TiO_2_-Pt photocatalyst was therefore evaluated in this study. The TiO_2_-Pt nanoparticles exhibited superior antibacterial activity compared to other commercial and laboratory-prepared photocatalysts in the *E. coli* experiments (Fig. 1). However, a major challenge is that soil pathogens can form biofilms, which can greatly reduce the antibacterial effect of some bactericides [33]–[35]. UV-responsive photocatalysts have been shown to eliminate the bacteria within a biofilm and suppress biofilm formation [41], [42]. However, whether a biofilm contributes to resistance against photocatalysis has not yet been ascertained. The degree to which visible light-responsive photocatalysts eliminate biofilm bacteria is also unclear. Using soil bacteria with differential biofilm-forming abilities, our data clearly demonstrate that biofilm formation is associated with resistance to photocatalysis (Table 1, Fig. 5–6, Fig. 6). The physical barrier created by a biofilm contributes to resistance against bactericides. The extracellular polysaccharide matrices of biofilms can restrict the diffusion and binding of soluble substances to the target bacteria, and thus the bacteria within the biofilm are protected [33]. This type of protection effectively resists the ROS hydrogen peroxide [36], [37]. Biofilm formation may play a more important role than catalase expression in resistance to H_2_O_2_ [36], [37]. A similar mechanism may be responsible for the resistance to the TiO_2_-Pt-photocatalysis-produced ROS in this study. Despite this protection, the TiO_2_-Pt photocatalyst still efficiently attenuated the pathogens.

Our mouse model revealed that the pathogenic potency of *B. pseudomallei* was greatly reduced after photocatalysis (Fig. 2). Because the injection dose of photocatalyzed cells was 1×10^3^ CFU/mouse and ultimately no mortality occurred after the injections (mortality occurred >1×10^1^ CFU/mouse; Fig. 2A and 2B, 1×10^1^ CFU groups; Fig. 2C, TiO_2_-Pt vs. no TiO_2_-Pt groups), it was estimated that at least a 2-log reduction in CFU was achieved (1×10^3^ to <1×10^1^ CFU). This is in agreement with our previous study that used photocatalyzed *B. anthracis* as a model [6]. In both systems, the plating method revealed a less than one-log reduction in viable cells (Table 1), while the *in vivo* experiments showed an additional reduction over one log (Fig. 2) [6]. Recently, photocatalysis was found to induce deformation and protein leakage in bacterial cells [9]. This damage is crucial for the antibacterial outcome; however, bacterial cells may be able to repair this damage and deformation [9]. Our results suggest that photocatalysis likely induced temporary, repairable damage to the bacteria, which could be recovered on the culture dishes (Table 1, *B. pseudomallei* vgh07 groups, 17% survived) but could not be recovered under phagocytic clearance *in vivo* (Fig. 2C, no mortality of TiO_2_-Pt groups, suggesting the survived bacteria <10^1^ CFU). In summary, visible light-responsive TiO_2_-Pt-mediated photocatalysis successfully attenuated soil-borne pathogens. In addition, the mouse mortality assay appears to be a relatively sensitive method for detecting sublethal damage to bacteria, compared to the traditional plating method.

An intriguing feature of the TiO_2_-Pt photocatalyst is its antibacterial performance in soil-contaminated water. When compared with bromophenol blue, soil contaminants seem to have only a limited effect on the inhibition of antibacterial photocatalysis (Fig. 7). This phenomenon has not been previously described. Soil particles contain various components with different surface-binding properties [43], [44]. Because photocatalyst-bacteria binding is critical for the antibacterial outcome of photocatalysis [7], soil particles may serve as bridging materials to link the bacteria and the TiO_2_-Pt nanoparticles and thus facilitate killing. However, a large amount of soil particles prohibits the filming of bacteria-photocatalyst aggregates. Moreover, soil can be penetrated by light to a certain extent, through which the seeds of certain plants are revived by light induction in particular seasons [45], [46]. Through pathways like these, the antibacterial performance of TiO_2_-Pt might be preserved. By contrast, bromophenol blue has been used widely in fluorescence quenching techniques in which fluorescent signals are greatly diminished by the addition of a dye [47], [48]. Such a mechanism might reduce the amount of light that reaches TiO_2_-Pt surfaces. Thus, bromophenol blue and soil particles likely have intrinsic differences in light absorption, scattering and reflection properties. The detailed mechanism underlining the differences in blocking of photocatalysis by dye and soil solutions is an interesting phenomenon that merits further investigation.

In summary, this study is the first to report that biofilm formation is associated with bacterial resistance to photocatalysis-mediated killing. Accordingly, biofilm formation may need careful evaluation and attention if photocatalysts are used to eliminate bacteria in biofilms. Nonetheless, nanoscale TiO_2_-Pt is an effective bactericide for eliminating soil-borne pathogens. The antibacterial effect significantly reduced the number of viable bacteria and damaged the residual surviving cells, achieving a greater than 2-log reduction in their estimated pathogenic potency. In addition, soil contaminants up to 10% w/v did not significantly reduce the antibacterial performance of TiO_2_-Pt. Titania photocatalysts have previously been applied as a suspension in a slurry UV reactor, as a thin film coated on a reactor surface or as a membrane filter [2], suggesting that TiO_2_ photocatalysts may be useful in a variety of settings to reduce the transmission of pathogens in public environments. Because specific methods to control the spread of these bacteria are still lacking, visible-light responsive TiO_2_-Pt photocatalysts may have potential applications in the development of effective antibacterial strategies against these soil-borne pathogens.

## Materials and Methods

### Ethics Statement

The animal methods in this study were approved by the Institutional Animal Care and Use Committee at the National Kaohsiung Normal University, Taiwan (approval ID: 9801 and 9901), and the experiments were performed in accordance with the institutional guidelines. These approvals certified the studies of the bactericidal activity of synthesized antibacterial compounds on the elimination of human pathogens, the air pouch infection mouse model and the pathophysiological observation of mouse sera or tissues after infection with *B. pseudomallei* and *B. cenocepacia.*

### Preparation of photocatalysts

Platinum-containing nano-structured TiO_2_ particles (TiO_2_-Pt) were prepared by the photoreduction process using chloroplatinic acid (H_2_PtCl_6_) and commercial TiO_2_ nanoparticles (Ishihara ST01) as a platinum precursor and a pristine photocatalyst, respectively. TiO_2_-Pt was prepared by mixing 3 g nonporous TiO_2_ (ST01) and 97 mg H_2_PtCl_6_·6H_2_O in 100 mL of double-distilled water. The TiO_2_ suspension and the H_2_PtCl_6_ solution were mixed well by ultrasonic treatment for 30 minutes. The initial pH value was adjusted to 4 with 0.1 M NaOH. A nitrogen stream at a rate of 100 mL/minute was continuously purged into the reaction chamber to remove oxygen in the solution. The solution was then irradiated with an UVC lamp (TUV 10W/G10 T8, Philips Taiwan, Taipei, Taiwan) with an intensity of 0.7 mW/cm^2^ for 4 hours. Platinum ions were reduced to platinum metallic nanoparticles by the photo-generated electrons of TiO_2_ and then deposited onto the surfaces of the TiO_2_. TiO_2_-Pt particles with a Pt/Ti molar ratio of 0.5% were obtained by centrifuging at 1×10^4^ rpm, washing with D.I. water and then drying at 373 K for 3 hours.

### Bacterial strains and culturing

*Staphylococcus aureus* BCRC10451, *Escherichia coli* BCRC11634, *Legionella pneumophilia* ATCC33152, *Klebsiella pneumoniae* nknu24 and *Salmonella typhimurium* FY148 were kindly obtained from the Center for Environmental Services (National Kaohsiung Normal University, Taiwan). *Burkholderia pseudomallei* (vgh07, vgh19 and vgh21), *Pseudomonas aeruginosa* FY32 and multidrug-resistant (Strain 49) and drug-susceptible *Acinetobacter baumannii* nknu11 were received from the Kaohsiung Veterans General Hospital (KVGH, Taiwan); their characteristics have been described in previous studies [49]–[51]. *Burkholderia cenocepacia* BC14 and other *Staphylococcus* strains (Strain 27, 41 and 69) were isolated from patients with septicemia in KVGH, Taiwan. All of the other *B. cenocepacia* strains (32 different isolates), including P2 and 34B, were isolated from the soil at the Er-Ren River Basin and the countryside of Kaohsiung County, Taiwan. All of the strains were confirmed by an automatic system (BD Phoenix 100 Automated Microbiology System, Becton, Dickinson and Company, Franklin Lakes, NJ) and, if necessary, by the nucleotide sequences of their 16S rRNA gene sequences. To avoid the light shedding effect, none of the *B. cenocepacia* strains used were pigment producers. Luria-Bertani (LB) broth was used to culture all of the bacteria.

### Detection of viable bacteria in suspensions or biofilms after photocatalysis

Bacterial concentrations were determined by the standard plating method [52], [53] and inferred from optical density readings at 600 nm (OD_600_). A factor for converting the OD_600_ values of the bacterial culture to concentration (colony forming units [CFU]/mL) was calculated as follows. A fresh bacterial culture was diluted by factors of 10^−1^ to 10^−7^, and the OD_600_ of these dilutions was measured. The bacterial concentrations of these dilutions were determined by the standard plating method. The OD_600_ values were plotted against the bacterial concentration log values, and the conversion factors for particular bacteria were calculated. The relative amount of viable bacteria estimated using this calculation was confirmed by the plating method.

To determine the bactericidal effects of the photocatalysts, 200 µl overnight bacterial culture was transferred into 5 mL culture medium and incubated at 37°C until an OD_600_ of 0.3 to 0.6 (log phase) was achieved. The bacterial concentrations were calculated using the conversion factor for the bacteria. Aliquots of 1×10^5^ CFU bacteria were mixed with TiO_2_-Pt nanoparticles (50 µg/mL) using a plastic yellow tip and placed onto a 24-well plate. For the photocatalytic reaction, the 24-well plates containing bacteria were then placed under an incandescent lamp (Classictone incandescent lamp, 60W, Philips Taiwan, Taipei, Taiwan); no UV range emissions of incident light were present. A light meter (LX-102, Lutron Electronic Enterprises, Taipei, Taiwan) was used to measure the illumination density. To compare the photocatalysis activity of TiO_2_-Pt with other photocatalysts, illuminations were performed with an illumination density of 1×10^4^ lux for 40 minutes. After illumination, the bacterial solutions were recovered, and an aliquot of fresh culture medium was used to collect the residual bacteria from the wells. These two bacterial solutions were pooled together. The bacterial concentration was determined by the standard plating method immediately after bacterial collection, and the percentage of surviving bacteria was calculated. A commercially available UV-responsive photocatalyst, ST01 (also the pristine photocatalyst), and a UV/Vis-responsive photocatalyst, BA-PW25 (Ecodevice, Tokyo, Japan) [28], [29], were used as comparisons.

Biofilm formation was performed on 96-well multiwell plates [54]. Suspensions of *B. cenocepacia* or *B. pseudomallei* (10^8^ CFU/mL; 100 µl) in log phase were seeded into polystyrene 96-well plates (BD Falcon, Erembodegem, Belgium). Four hours post-adhesion, the non-adhered cells were removed using 100 µL normal saline (0.9% w/v NaCl). After 3 washes, the photocatalytic reaction was conducted in the 96-well plates, which contained adherent bacterial cells (approximately 1×10^5^ CFU) and 20 µg TiO_2_-Pt in 0.5 mL saline, with illumination with visible light with a density of 4×10^4^ lux. To measure the relative masses of the respective biofilms, 100 µL fresh LB broth was added to each well after washing with normal saline in another plate. After incubation for 20 hours, the supernatant was again removed, and the wells were washed with 100 µL saline solution. In the crystal violet assay, 100 µL 99% methanol was added and incubated for 15 minutes to fix the biofilms, after which the supernatants were removed and the plates were air-dried. Next, 100 µL crystal violet solution (0.1%, Pro-Lab Diagnostics, Richmond Hill, ON, Canada) was added to each well. After 20 minutes at room temperature, the excess crystal violet was removed by washing the plates under running tap water. Finally, the bound crystal violet was released by adding 150 µL 33% acetic acid (Sigma-Aldrich, St. Louis, MO). The absorbance was measured at 590 nm using a multilabel microtiter plate reader (Wallac Victor; Perkin Elmer Life and Analytical Sciences, Boston, MA).

### Mouse mortality analysis

To determine the lethal doses of *B. pseudomallei* vgh07 and *B. cenocepacia* P2, the bacteria were grown to log phase (OD_600_ of 0.5–0.6). After 3 washes and resuspension with normal saline, the bacteria solutions were diluted to various concentrations (1×10^1^ to 1×10^7^ CFU in 0.5 mL saline). BALB/cJ mice then received intravascular treatments with these bacteria (1×10^1^ to 1×10^7^ CFU/mouse), and their mortality was recorded every day for 6 days. To evaluate photocatalysis-mediated attenuation, 1×10^3^ CFU *B. pseudomallei* (log phase) were treated with or without photocatalysis (20 µg TiO_2_-Pt, 1×10^4^ lux, 40 minutes, in 100 µL saline). The mortality was recorded after the mice were treated with the bacteria intravascularly.

### Hematoxylin and eosin (H & E) staining

After treatment with the photocatalyzed bacteria for 24 hours, livers were excised from the infected mice, fixed in 10% formalin, embedded in paraffin, sectioned (4 µm) and stained with hematoxylin and eosin. The images of necrosis and cellular debris in the liver tissue were observed in 30 high-power fields (400×) under a microscope (DMIRE2; Leica, Wetzlar, Germany).

### Analysis of liver function

To analyze liver function, whole blood samples (50–100 µl) were collected from mice hearts and mixed with anticoagulant solution (0.1 M sodium citrate) in Eppendorf tubes. The levels of aspartate aminotransferase/alanine aminotransferase (AST/ALT) were measured with a clinical biochemistry analysis system (COBAS INTEGRA® 800, Roche Taiwan, Taipei, Taiwan) at 24 hours post-infection.

### Detection of LPS

The concentration of 3-hydroxytetradecanoic acid (C14:0 3-OH FA), a surrogate for LPS, was measured with an Agilent 6890 gas chromatograph/5973N mass selective detector (GC-MS) system [55]. Briefly, the 7-day-cultures of each strain were filtered (0.45 µm), lyophilized and resuspended in 1 ml methanolic NaOH (3.8N). This methanolic solution was heated to 100°C in a cooled counterflow system for 30 minutes and then adjusted with methanolic HCl (2.5N) at 80°C for another 10 minutes. After esterification, hexane was added as a partition solution in a proportion of 1∶1 for 5 minutes. The upper organic aqueous layer was removed, dried with nitrogen gas and resuspended using 1 ml of hexane.

The GC was equipped with a 60-m DB23-MS (Andover, MA) capillary column (0.25-mm ID; 0.25-µm film thickness). The injector and interface temperatures were maintained at 260°C and 280°C, respectively. The oven temperature was held at 110°C for 1 minute, and then a two-step program was used to increase the temperature to 175°C at 25°C/minute and then to 220°C at 1.5°C/minute. Finally, the temperature was held at 220°C for 3 minutes. The following parameters were used for injecting the samples into the GC/MSD system: sample size, 1 µL; injection mode, splitless; injector purge-off duration, 1 minute; solvent delay, 5.9 minuts.

The mass range adopted for the collection of the full-scan mass spectra was m/z 50–550. Based on the ion intensity data from the full-scan mass spectra, the differentially fragmented ions (43/103/166 m/z) were then used for identification and quantification. The amount of 3-OH FA present, after adjustment relative to the recovered internal standard concentration, was plotted against the ratio of the areas of the tested C14:0 3-OH FA and the areas of the standards.

### Detection of cytokines

TNF-α and IL-1 levels in the mouse sera were analyzed using ELISA kits (Diaclone Inc., Besancon Cedex, France) according to the manufacturer's instructions.

### Scanning electron microscopic (SEM) imaging

The SEM analysis was performed as previously described [56]. Photocatalyzed bacteria were incubated on bovine serum albumin-precoated (1% w/v) cover slides for 15 minutes. Bacteria that were attached to the cover slides were then fixed with glutaraldehyde and subjected to alcohol dehydration, critical point drying procedures, and gold coating [57] and observed under a scanning electron microscope at 15 kV (S-4700, Hitachi High-Technologies, Tokyo, Japan). At least three different areas were randomly selected for photography at each magnification; representative data are shown.

### Air pouch infection mouse model

BALB/cJ mice (males, 8–10 weeks of age) were purchased from the National Laboratory Animal Center (NLAC, Taipei, Taiwan). The mice were housed in the Laboratory Animal Center of National Kaohsiung Normal University (Kaohsiung, Taiwan). The air pouch infection was modified based on a previously described approach [8], [32]. After anesthesia and shaving of the hairs around the injection sites, the BALB/cJ mice were subcutaneously injected with 1 mL air to form an air pouch. Suspensions of *B. cenocepacia* (log phase, 10^5^ CFU, 0.2 mL phosphate buffered saline [PBS]) with or without photocatalysis were injected into the air pouches. At 48 hours after photocatalytic treatment, the air pouches were injected with an additional 1 mL PBS. The bacteria-containing solution (1 mL) was then collected from the air pouch. The number of surviving bacteria (CFU) was determined using the standard plating method.

### Photocatalysis in bromophenol blue- and soil-containing solutions

The soil samples were obtained from the Er-Ren River Basin in southern Taiwan, which features vigorously growing vegetation and is a natural habitat for *B. pseudomallei* and *B. cenocepacia* [22]. After dissolution in distilled water, the large particles were removed using a one-mm sieve. The remaining small particles were sterilized using an autoclave. After sedimentation by centrifugation at 600 g for 10 minutes, the soil particles were resuspended in normal saline. Bacteria (1×10^5^ CFU) and the photocatalyst-containing saline (20 µg TiO_2_-Pt) solutions were supplemented by various concentrations of bromophenol blue (Sigma-Aldrich) or soil particles to a final volume of 0.5 mL saline solution. Photocatalysis was performed in 24-well dishes with illumination of visible light at a density of 4×10^4^ lux. The viable bacteria were then determined using the plating method.

### Determination of the specific growth rate constant of B. cenocepacia P2

*B. cenocepacia* P2 was grown in LB, BCS (*B. cepacia* selective) or soil media. The specific growth constant (K) was calculated during the first 3 to 5 hours of growth at 37°C with the equation lnN-lnN_0_ = K (t-t_0_) and was derived from the mean of triplicate experiments. N represents the cell concentration per mL of a five-hour culture (t); N_0_ represents the cell concentration of a three-hour culture (t_0_) [58].

### Statistical analysis

All of the results were calculated using data from at least three independent experiments. The *T*-test was used to assess the statistical significance of differences in antimicrobial effects. A *P* value of less than 0.05 (*P*<0.05) was considered significant. The statistical tests were performed and output to graphs using Microsoft Excel (Microsoft Taiwan, Taipei, Taiwan) and SigmaPlot (Systat Software, Point Richmond, CA).
