# Correction: The Use of Nanoscale Visible Light-Responsive Photocatalyst TiO_2_-Pt for the Elimination of Soil-Borne Pathogens

**DOI:** 10.1371/annotation/833343b8-c3a7-4dc2-9b85-578188a1590a

**Published:** 2012-04-09

**Authors:** Ya-Lei Chen, Yao-Shen Chen, Hao Chan, Yao-Hsuan Tseng, Shu-Ru Yang, Hsin-Ying Tsai, Hong-Yi Liu, Der-Shan Sun, Hsin-Hou Chang

There was an error in the funding statement that was introduced during the production process. The correct funding statement is: This work is supported by the National Science Council, Taiwan, Republic of China, under grant numbers 95-2314-B-320-009-MY3, 98-2320-B-0.17-001-MY3 and 99-2320-B-017-002 -MY3, by the Ministry of Economic Affairs, Taiwan, Republic of China, under grant number 98-EC-17-A-19-S2-0111 and by Tzu-Chi University under grant numbers TCIRP 95002-02, TCIRP 98001-01, TCRPP 99020 and TCRPP 100003. The funders had no role in study design, data collection and analysis, decision to publish, or preparation of the manuscript. 

